# Supplementary figures and images for: Distribution, evolution and expression of GATA-TFs provide new insights into their functions in light response and fruiting body development of Tolypocladium guangdongense
Source: PeerJ. 2020 Aug 28;8:e9784. doi: 10.7717/peerj.9784 (PMC7457929; doi:10.7717/peerj.9784)

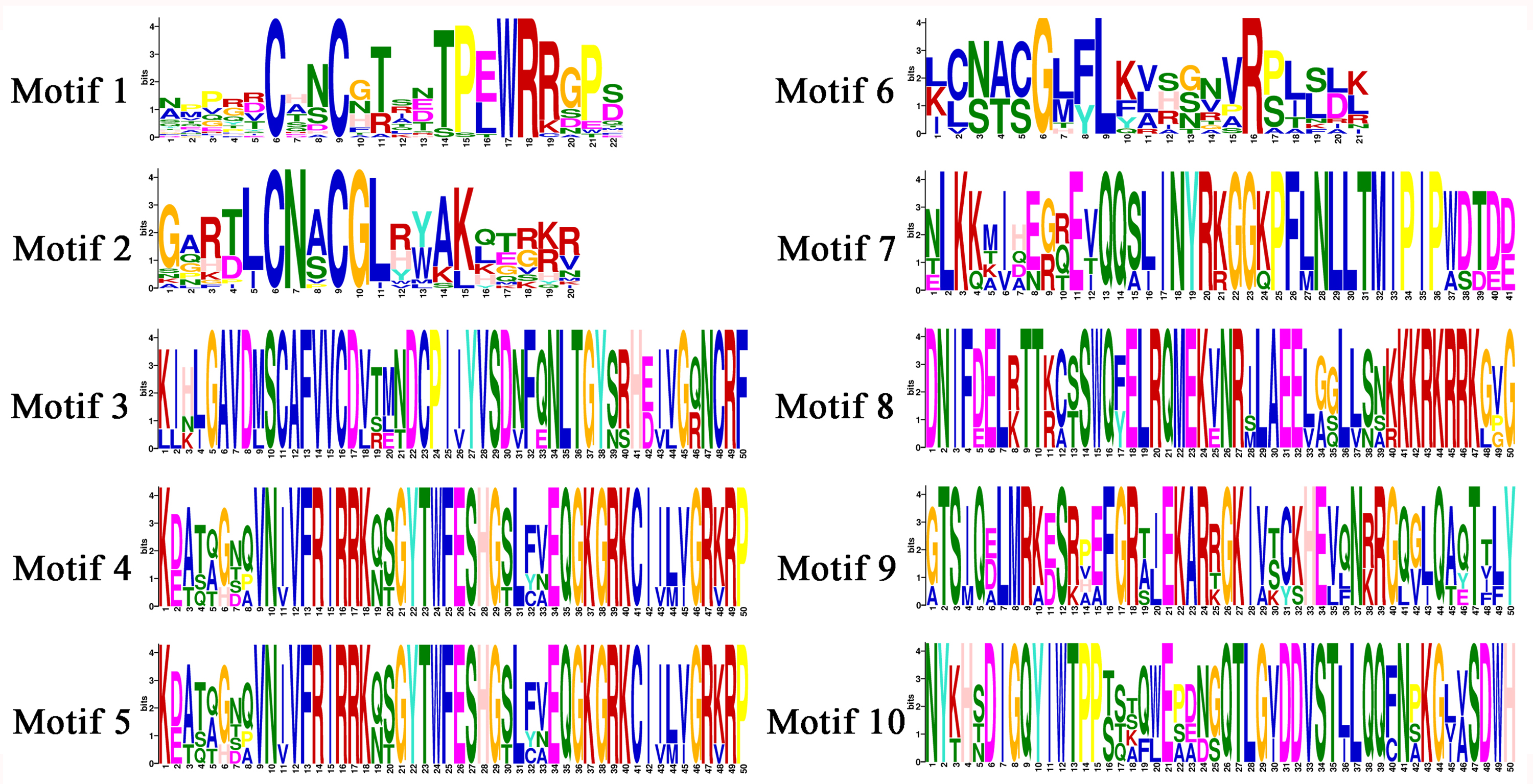

Supplement: Figure S1 [file peerj-08-9784-s010.jpg]

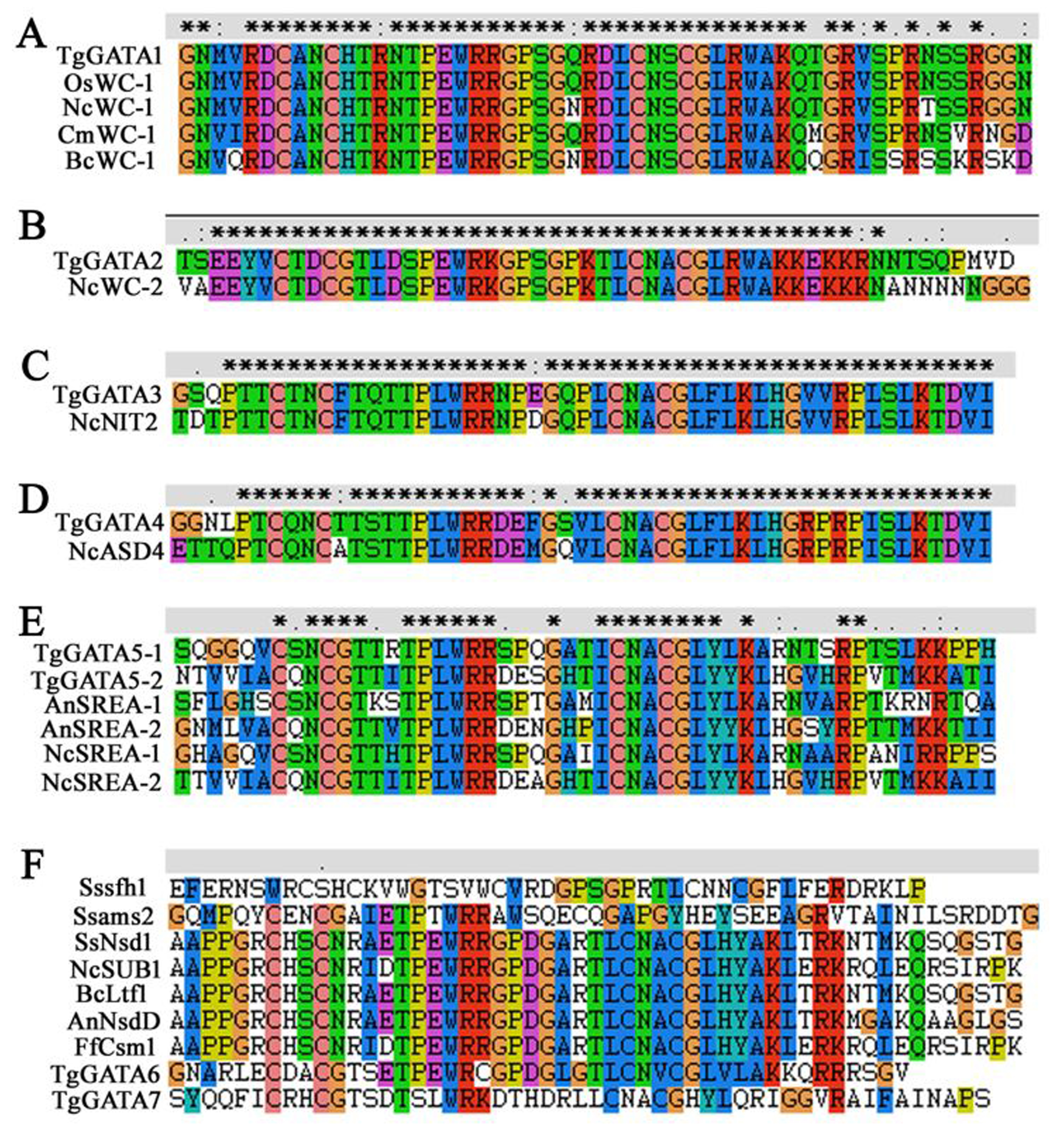

Supplement: Figure S2 [file peerj-08-9784-s011.jpg]
